# ADORA2A activation restores lysosomal function and photoreceptor outer segment degradation in stressed retinal pigment epithelium

**DOI:** 10.3389/fphys.2026.1866784

**Published:** 2026-08-05

**Authors:** Daodian Tao, Daiying Zhou, Mingjuan Wu, Peiling Xie, Yunhao Zhang, Jing Bao

**Affiliations:** 1 Department of Ophthalmology, Jinhua Municipal Central Hospital, Jinhua, China; 2 Department of Ophthalmology, Sichuan Provincial People’s Hospital East Sichuan Hospital&Dazhou First People’s Hospital, Dazhou, China; 3 Department of Health Management Center, Jinhua Municipal Central Hospital, Jinhua, China; 4 Department of Wushu and Traditional Chinese Ethnic Sports, Wuhan Sport University, Wuhan, China

**Keywords:** ADORA2A, age-related macular degeneration, lysosomal acidification, post-ingestive degradation, retinal pigment epithelium

## Abstract

**Background:**

Age-related macular degeneration (AMD) involves early retinal pigment epithelium (RPE) dysfunction and impaired processing of photoreceptor outer segments (POS). We investigated whether ADORA2A regulates post-ingestive POS handling and lysosomal recovery under AMD-relevant stress.

**Methods:**

A2E-stressed ARPE-19 cells, primary porcine RPE cells, and a sodium iodate-induced mouse model were studied. ADORA2A was activated with CGS21680 and inhibited with ZM241385. POS handling, LC3B/Rubicon association with POS, lysosomal function, Rubicon depletion, chronic stress phenotypes, and retinal protection were assessed.

**Results:**

A2E increased ADORA2A expression while preserving receptor-dependent cAMP responsiveness. CGS21680 had modest effects on early POS binding and uptake but enhanced post-ingestive POS clearance and rhodopsin degradation; these effects were attenuated by ZM241385 and ADORA2A knockdown. CGS21680 increased LC3B and Rubicon association with POS-containing structures, improved lysosomal acidification, and restored DQ Green BSA processing, Cathepsin D activity, and V-ATPase-associated assembly. Bafilomycin A1 increased LC3-II and p62 accumulation and impaired CGS21680-associated POS clearance, supporting lysosome-dependent turnover. Rubicon depletion attenuated CGS21680-associated improvements in POS clearance and lysosomal acidification. CGS21680 also reduced chronic stress-associated autofluorescence, oxidative stress, apoptosis, and junctional disruption. These protective effects were reproduced in primary RPE cells. In sodium iodate-injured mice, CGS21680 preserved outer retinal structure, reduced FITC-BSA leakage, improved electroretinographic responses, and was accompanied by increased CREB phosphorylation and recovery of Cathepsin D proteolytic competence.

**Conclusion:**

ADORA2A activation promotes Rubicon-associated, lysosome-dependent LC3 processing of internalized POS and restores lysosomal degradative competence, supporting ADORA2A as a potential therapeutic target for early AMD.

## 1 Introduction

Age-related macular degeneration is a degenerative disorder of the macula and a leading cause of central vision loss in older adults (Fleckenstein et al., 2024). Although late-stage AMD is clinically defined by choroidal neovascularization or geographic atrophy, disease progression begins much earlier (Mitchell et al., 2018; Curcio et al., 2024). Increasing evidence supports a model in which AMD arises from chronic disequilibrium across the photoreceptor–retinal pigment epithelium–Bruch’s membrane–choriocapillaris unit, with RPE dysfunction emerging as an early and persistent pathogenic event (Bhutto and Lutty, 2012; Guymer and Campbell, 2023). In this context, RPE failure is not simply a downstream consequence of retinal degeneration (Kim et al., 2021). Rather, it is increasingly viewed as a central driver of disease onset and progression because the RPE sits at the interface of photoreceptor support, outer retinal metabolism, and local tissue homeostasis. Once this support system begins to fail, defects in nutrient handling, redox balance, barrier integrity, and waste clearance can converge to destabilize the outer retina long before overt end-stage lesions appear (Somasundaran et al., 2020; Hansman et al., 2025). Recent reviews have increasingly highlighted aged and stressed RPE cells as a major early pathogenic hub in AMD (Hansman et al., 2025).

A major reason for the central role of the RPE is its continuous responsibility for phagocytosis and degradation of photoreceptor outer segments (Lakkaraju et al., 2020). Under physiological conditions, POS uptake is coupled to phagosome maturation, lysosomal fusion, acidification, and proteolysis, forming a tightly integrated degradative pathway (Kwon and Freeman, 2020; Zhang et al., 2024). This pathway is especially vulnerable to ageing, bisretinoid accumulation, photo-oxidative stress, and chronic metabolic burden. A2E and lipofuscin-associated stress are closely linked to lysosomal dysfunction, impaired degradative capacity, and progressive RPE injury (Jiménez-Loygorri et al., 2025). The consequence is progressive accumulation of incompletely cleared lipids and proteins, with secondary amplification of reactive oxygen species, inflammatory signaling, and cellular dysfunction (Senabouth et al., 2022; Karema-Jokinen et al., 2023). These observations have shifted attention from POS uptake alone to the broader concept of phagolysosomal fitness, whether engulfed cargo can still be processed efficiently after internalization (Ma et al., 2023). Recent work has continued to link oxidative stress and lysosomal dysfunction in the RPE to early AMD-related degeneration, underscoring that impaired post-phagocytic degradation is likely a core lesion rather than a secondary epiphenomenon.

How the RPE handles POS has also been redefined over the past decade. POS processing does not appear to rely solely on canonical autophagy, but instead shows features of LC3-associated phagocytosis, a non-canonical pathway in which LC3 is recruited to single-membrane phagosomes and facilitates maturation and degradation (Saito et al., 2020). In RPE cells, Rubicon has been implicated in this process and is required for efficient post-phagocytic degradation of outer segments. Enhancement of LC3-associated POS processing can improve cargo handling, supporting the idea that LAP-like mechanisms contribute materially to outer-segment clearance in the RPE (Muniz-Feliciano et al., 2017; Saito et al., 2020; Kaarniranta et al., 2023). At the same time, the contribution of LAP-like processing under AMD-relevant stress conditions remains unresolved. It is not yet clear whether this pathway is itself impaired in stressed RPE cells, how much it contributes to the decline in degradative capacity, or whether restoring it is sufficient to improve cargo clearance in a biologically meaningful way. These questions are important because they place the problem of POS handling at the intersection of autophagy-related machinery, phagosome maturation, and lysosomal competence, rather than within any one of these processes alone (Intartaglia et al., 2022; Pinelli et al., 2025).

Adenosine signaling offers a plausible upstream entry point into this problem. RPE cells express A2-type adenosine receptors, and receptor activation increases intracellular cAMP (Friedman et al., 1989). In models of lysosomal dysfunction, including A2E-loaded RPE cells, cAMP elevation or A2A receptor stimulation has been shown to re-acidify lysosomes and enhance post-phagocytic degradative processing, although its effect on early POS ingestion appears to be context dependent (Guha et al., 2014; Goebel et al., 2023). These observations place ADORA2A–cAMP signaling as a candidate regulator of degradative competence in stressed RPE cells (Liu et al., 2008). However, existing evidence has focused mainly on lysosomal pH and proteolysis. Whether this pathway also influences LC3 recruitment to POS-containing phagosomes, and thereby links stress responses to LAP-like post-phagocytic processing, has not been established. Thus, an important unresolved question in AMD is whether a common upstream pathway connects stress sensing, defective POS handling, and impaired post-phagocytic degradation in the RPE (Liu et al., 2025). Here, we asked whether ADORA2A–cAMP signaling contributes to AMD-related RPE dysfunction by coordinating POS processing with post-phagocytic degradative remodeling. Defining this pathway may also inform the search for mechanism-based targets for early intervention.

## 2 Materials and methods

### 2.1 Reagents and antibodies

A2E (pyridinium bisretinoid A2E, trifluoroacetate salt; HY-134928A) was purchased from MedChemExpress. CGS21680 hydrochloride (1063), ZM241385 (1036), and H-89 dihydrochloride (2910) were purchased from Tocris Bioscience. GSK2795039 (SML2770), E64d (E8640), and pepstatin A (P5318) were purchased from Sigma–Aldrich. LysoSensor Yellow/Blue DND-160 (L7545), DQ Green BSA (D12050), Texas Red-X succinimidyl ester (T6134), Trypan Blue solution (0.4%; T10282), Lipofectamine RNAiMAX (13778075), and Lipofectamine 3000 (L3000008) were obtained from Thermo Fisher Scientific. The Cathepsin D Activity Assay Kit was obtained from Abcam (ab65302). The Epac-S-H187 cAMP biosensor plasmid was obtained from Addgene (#170348). Primary antibodies used in this study were ADORA2A (Abcam, ab3461), phospho-CREB (Ser133) (Cell Signaling Technology, 9198), CREB (Cell Signaling Technology, 9197), LC3B (Cell Signaling Technology, 3868), SQSTM1/p62 (Cell Signaling Technology, 5114), Rubicon (Proteintech, 21444-1-AP), STX17 (Cell Signaling Technology, 31261), ATP6V1A (Proteintech, 68440-1-Ig), ATP6V0A1 (Proteintech, 13828-1-AP), Cathepsin D (Abcam, ab75852), ZO-1 (Proteintech, 21773-1-AP), phospho-PKA substrate (RRXS/T) (Cell Signaling Technology, 9624), GAPDH (Proteintech, 66009-1-Ig).

### 2.2 Animals

Male C57BL/6 mice aged 8–10 weeks and weighing 20–24 g were obtained from Cyagen Biosciences Inc. (Suzhou, China). Mice were housed under specific pathogen-free conditions at 22 ± 2 °C, with a 12-h light/dark cycle, 50%–60% relative humidity, and free access to food and water. All mice were acclimatized for at least 1 week before the experiments. All animal procedures were approved by the institutional animal care and use committee and were performed in accordance with institutional guidelines(AL-JHYY202644).

To induce RPE degeneration, mice received a single intraperitoneal (i.p.) injection of sterile sodium iodate (NaIO3, Sigma-Aldrich, St. Louis, MO, USA) dissolved in 0.9% saline at a dose of 25 mg/kg. Control mice received an equivalent volume of 0.9% saline. For pharmacological interventions, the ADORA2A agonist CGS21680 (1.0 mg/kg, dissolved in 0.9% saline containing 1% DMSO; Sigma-Aldrich) or vehicle control was administered via daily i.p. injections starting 1 day prior to $NaIO_3$ administration and continuing for 7 consecutive days. To determine receptor specificity *in vivo*, an additional cohort of mice was co-treated with the selective ADORA2A antagonist ZM241385 (3.0 mg/kg, dissolved in a vehicle of 5% DMSO and 5% Tween-80 in 0.9% saline; Sigma-Aldrich). ZM241385 was administered i.p. 30 minutes prior to each CGS21680 injection throughout the treatment period.

### 2.3 Cell culture

Human retinal pigment epithelial cells (ARPE-19) were purchased from the Cell Bank of the Type Culture Collection of the Chinese Academy of Sciences (Shanghai, China). The cells were propagated in DMEM/F12 medium supplemented with 10% fetal bovine serum and 1% penicillin-streptomycin. Cultures were maintained at 37 °C in a humidified incubator with a 5% CO_2_ atmosphere. For experiments involving phagocytosis or lysosomal function, ARPE-19 cells were seeded onto sterile glass coverslips or multi-well culture plates and allowed to grow until they reached complete confluence prior to any treatment.

### 2.4 Dark-adapted electroretinography analysis

Prior to electroretinography (ERG) evaluation, mice were dark-adapted overnight. Animals were anesthetized by intraperitoneal injection of a ketamine (87.5 mg/kg) and xylazine (12.5 mg/kg) mixture. The corneas were topically anesthetized with 0.5% proparacaine hydrochloride, and the pupils were dilated with 2.5% phenylephrine hydrochloride. Mice were then placed on a temperature-regulated heating platform (Diagnosys LLC, Lowell, MA) to maintain core body temperature. Retinal responses were recorded by placing gold ring electrodes on each cornea; a subdermal reference electrode was positioned on the nose, and a ground needle electrode was placed at the base of the tail. Full-field scotopic ERG responses were recorded using the Espion system (Diagnosys LLC). To evaluate outer retinal and RPE function, flash intensities were set at 0.01, 0.1, and 1 cd·s/m² (corresponding to −2, −1, and 0 log cd·s/m², respectively). The amplitude of the a-wave was measured from the pre-stimulus baseline to the trough of the first corneal negative deflection. The b-wave amplitude was calculated from the a-wave trough to the peak of the b-wave. The c-wave was determined as the peak of the slow positive wave following the b-wave. All ERG a-, b-, and c-wave amplitudes were automatically calculated by Espion V6 software.

### 2.5 Permeability assay to measure vascular leakage

Leakage of albumin into the retina was evaluated using a FITC-BSA tracer assay. Briefly, mice were anesthetized via isoflurane and intravenously injected with FITC-BSA (100 mg/kg body weight). After 1 h of circulation, the mice were euthanized for subsequent analyses. For qualitative morphological visualization, eyes were rapidly enucleated, and the retinas were carefully dissected, flat-mounted, and imaged using a fluorescence microscope to observe extravascular dye leakage. For quantitative fluorometric analysis, whole blood was obtained from the right ventricle and centrifuged at 2000 × g for 15 min to isolate plasma, which was subsequently diluted 100-fold with PBS. Next, the mice were perfused via the left ventricle with PBS to completely remove intravascular blood. Whole retinas were isolated carefully to avoid contamination from the aqueous humor, homogenized in RIPA lysis buffer, and centrifuged at 16,600 × g for 15 min. The fluorescence intensities of the retinal homogenate supernatants and diluted plasma were measured using a Synergy H1 Hybrid Multi-Mode Reader (BioTek, Winooski, VT) with excitation at 485 nm and emission at 528 nm. Retinal homogenates and diluted plasma from mice without FITC-BSA injection were used as blanks. Finally, the retinal fluorescence intensity was adjusted by retinal weight and corresponding plasma fluorescence, and the final values were normalized to the control (CTRL) group.

### 2.6 Lysosomal stress assay

Confluent ARPE-19 cells were incubated with A2E at 10 μM for 48 h. For oxidative stress experiments, cells were treated with H_2_O_2_ at 200 μM for 24 h. CGS21680 was used at 100 nM. ZM241385 and H-89 were each used at 10 μM and were added 30 min before CGS21680 treatment. GSK2795039 was used at 10 μM and was added 30 min before CGS21680 treatment or POS challenge where indicated. For phospho-CREB and cAMP measurements, cells were exposed to CGS21680 for 15–30 min. For lysosomal pH, DQ Green BSA, Cathepsin D, and cargo-processing assays, compounds remained in the medium for the full assay period unless otherwise stated.

### 2.7 siRNA transfection

ARPE-19 cells were transfected with Silencer Select siRNA targeting human ADORA2A or negative control siRNA using Lipofectamine RNAiMAX according to the manufacturer’s instructions. The final siRNA concentration was 5 nM. Cells were collected 72 h after transfection for immunoblotting, quantitative PCR, immunofluorescence, and functional assays. For cAMP imaging, cells were transfected with Epac-S-H187 using Lipofectamine 3000 and analyzed 24–48 h later.

### 2.8 POS labeling

Commercial bovine rod outer segments were purchased from InVision BioResources (Rod Outer Segment (ROS), Bovine; catalog number 98740). ROS were labeled with Texas Red-X succinimidyl ester at 1 mg/ml in 0.1 M sodium bicarbonate (pH 8.5) for 1 h in the dark, washed extensively, and resuspended in culture medium containing 2.5% sucrose before use.

### 2.9 cAMP FRET assay

ARPE-19 cells transfected with Epac-S-H187 were washed with HEPES-buffered imaging medium and transferred to a live-cell imaging chamber. Baseline fluorescence was recorded for 3–5 min. CGS21680 was then added in the presence or absence of ZM241385 or H-89 pretreatment. Images were acquired every 10–20 s for 10 min using a fluorescence microscope configured for CFP/YFP FRET measurements. The cAMP response was quantified as the acceptor/donor fluorescence ratio and normalized to the baseline signal of each cell.

### 2.10 POS uptake assay

ARPE-19 monolayers were exposed to Texas Red-conjugated photoreceptor outer segments (POS) at a particle-to-cell ratio of 10:1 for 6 hours at 37 °C. Following incubation, unbound POS were removed by washing with PBS containing 1 mM MgCl_2_ and 0.2 mM CaCl_2_. To eliminate extracellular fluorescence, cells were incubated with 0.2% trypan blue for 10 minutes before fixation. Fluorescent images were captured using a confocal laser scanning microscope. Total POS and internalized POS were quantified with Fiji/ImageJ software.

### 2.11 POS pulse-chase degradation assay

ARPE-19 cells were fed unlabeled POS at a 10:1 POS:cell ratio for 6 h. After extensive washing to remove unbound POS, cells were incubated in POS-free medium for 16 or 24 h. Where indicated, E64d and pepstatin A were each added at 10 μM immediately after the pulse and maintained for 10 h before sample collection. Degradation of engulfed POS was assessed by immunoblotting and fluorescence-based cargo clearance readouts.

### 2.12 Immunofluorescence staining

Cells grown on coverslips were fixed with 4% paraformaldehyde for 15 min at ambient temperature, then permeabilized with 0.2% Triton X-100 in PBS. Non-specific binding was blocked by incubating the cells for 1 h at room temperature in PBS containing 5% goat serum and 0.1% Triton X-100. Primary antibodies directed against LC3B or Rubicon were applied overnight at 4 °C, followed by Alexa Fluor-conjugated secondary antibodies for 1–2 h at room temperature. Nuclei were counterstained with DAPI. For colocalization studies of LC3 with POS or Rubicon with POS, cells were challenged with Texas Red-labeled POS and fixed at 15, 30, or 60 min post-challenge. All images were acquired on a confocal microscope using identical acquisition settings within each experiment. For each independent experiment, at least 20 cells per condition were analyzed.

### 2.13 Lysosomal pH assay

Cells were incubated with LysoSensor Yellow/Blue DND-160 at 2 μM for 5–10 min at 37 °C, washed once with dye-free buffer, and imaged immediately. Dual-emission fluorescence images were acquired, and lysosomal pH was calculated from calibration curves generated with ionophore-containing buffers of defined pH.

### 2.14 DQ Green BSA assay

Cells were incubated with DQ Green BSA at 10 μg/ml for 2 h at 37 °C, washed in PBS, and imaged live or after fixation. Fluorescence intensity generated by proteolytic dequenching was quantified per cell after background subtraction and normalized to nuclear number.

### 2.15 Cathepsin D activity assay

Cathepsin D enzymatic activity was measured using a fluorometric assay kit (Abcam, ab65302) following the manufacturer’s protocol. Equal amounts of total protein lysate were incubated with the provided substrate for 1 h at 37 °C. Fluorescence intensity was read on a microplate reader at excitation/emission wavelengths of 328/460 nm. In parallel, the maturation status of cathepsin D was assessed by western blotting using an anti-cathepsin D antibody.

### 2.16 V-ATPase assembly assay

Lysosome-enriched fractions were prepared using the Lysosome Enrichment Kit for Tissue and Cultured Cells (Thermo Fisher Scientific, 89839) according to the manufacturer’s instructions. Samples were solubilized under non-denaturing conditions and separated by native PAGE. Proteins were transferred to PVDF membranes and probed with antibodies against ATP6V1A and ATP6V0A1. Band intensity was quantified by densitometry.

### 2.17 Western blotting

Cells were lysed in RIPA buffer fortified with protease and phosphatase inhibitor cocktails. Protein concentrations were determined via the bicinchoninic acid (BCA) method. Equal protein amounts (20 μg per lane) were resolved on 10–12% Bis-Tris polyacrylamide gels and electrotransferred onto PVDF membranes. Membranes were blocked with either 5% non-fat dry milk or 5% bovine serum albumin, then incubated overnight at 4 °C with primary antibodies against ADORA2A, phospho-CREB, total CREB, LC3B, SQSTM1/p62, Rubicon, ATP6V1A, ATP6V0A1, cathepsin D, ZO-1, or GAPDH. Horseradish peroxidase-linked secondary antibodies were applied for 1 h at room temperature. Immunoreactive bands were visualized using enhanced chemiluminescence and quantified with ImageJ. LC3-I and LC3-II were quantified as separate bands.

### 2.18 Quantitative real-time PCR

Total RNA was extracted using TRIzol reagent. First-strand cDNA was synthesized from 1 μg of RNA using a reverse transcription kit according to the manufacturer’s guidelines. Quantitative PCR amplifications were carried out on a QuantStudio real-time PCR system using SYBR Green chemistry. Relative mRNA levels of ADORA2A, RUBCN, and CTSD were normalized to the housekeeping gene GAPDH and calculated by the 2^−^ΔΔCt method.

### 2.19 Immunoprecipitation and co-immunoprecipitation

Cells were lysed in NP-40 lysis buffer containing phosphatase inhibitors. Equal amounts of lysate were incubated overnight at 4 °C with anti-Rubicon antibody, followed by incubation with Protein A/G magnetic beads. Immunoprecipitates were washed, eluted in SDS sample buffer, and analyzed by immunoblotting using phospho-PKA substrate antibody. For co-immunoprecipitation, Rubicon was immunoprecipitated and the precipitates were probed for VPS34 and NOX2. Input controls corresponding to 5–10% of total lysate were analyzed in parallel.

### 2.20 Intracellular ROS assay

Intracellular ROS was measured using CellROX Deep Red according to the manufacturer’s instructions. Cells were incubated with the probe for 30 min at 37 °C, washed with PBS, and analyzed by flow cytometry. Mean fluorescence intensity was normalized to cell number.

### 2.21 Cellular autofluorescence assay

Confluent ARPE-19 monolayers were challenged with POS once daily for 14 consecutive days. During the same period, cells were exposed to low-dose H_2_O_2_ to maintain sublethal oxidative stress. Cellular autofluorescence was analyzed by flow cytometry in the FITC channel after exclusion of debris and dead cells. Mean autofluorescence intensity was calculated for each group.

### 2.22 Annexin V/propidium iodide assay

Apoptosis and necrosis were evaluated by Annexin V-FITC/propidium iodide (PI) double staining following the kit instructions. After treatment, cells were harvested, washed with ice-cold PBS, resuspended in binding buffer, and stained with Annexin V-FITC and PI. Samples were analyzed by flow cytometry within 1 h of staining. Early apoptotic cells were identified as Annexin V-positive/PI-negative, whereas late apoptotic or necrotic cells were defined as Annexin V-positive/PI-positive.

### 2.23 Statistical analysis

All data are presented as mean ± standard error of the mean (s.e.m.) derived from at least three independent experiments, unless otherwise indicated. Statistical evaluations were performed using GraphPad Prism 10. Normality of the data was verified by the Shapiro–Wilk test. For comparisons between two groups, either a two-tailed unpaired Student’s t-test or the Mann–Whitney U test was applied as appropriate. For multiple group comparisons, one-way or two-way analysis of variance (ANOVA) followed by Tukey’s or Sidak’s post-hoc test was used. When data deviated from normal distribution, the Kruskal–Wallis test with Dunn’s correction was employed. A p-value < 0.05 was considered statistically significant.

## 3 Results

### 3.1 ADORA2A is induced by A2E stress and remains functionally responsive in RPE cells

**Figure 1:**
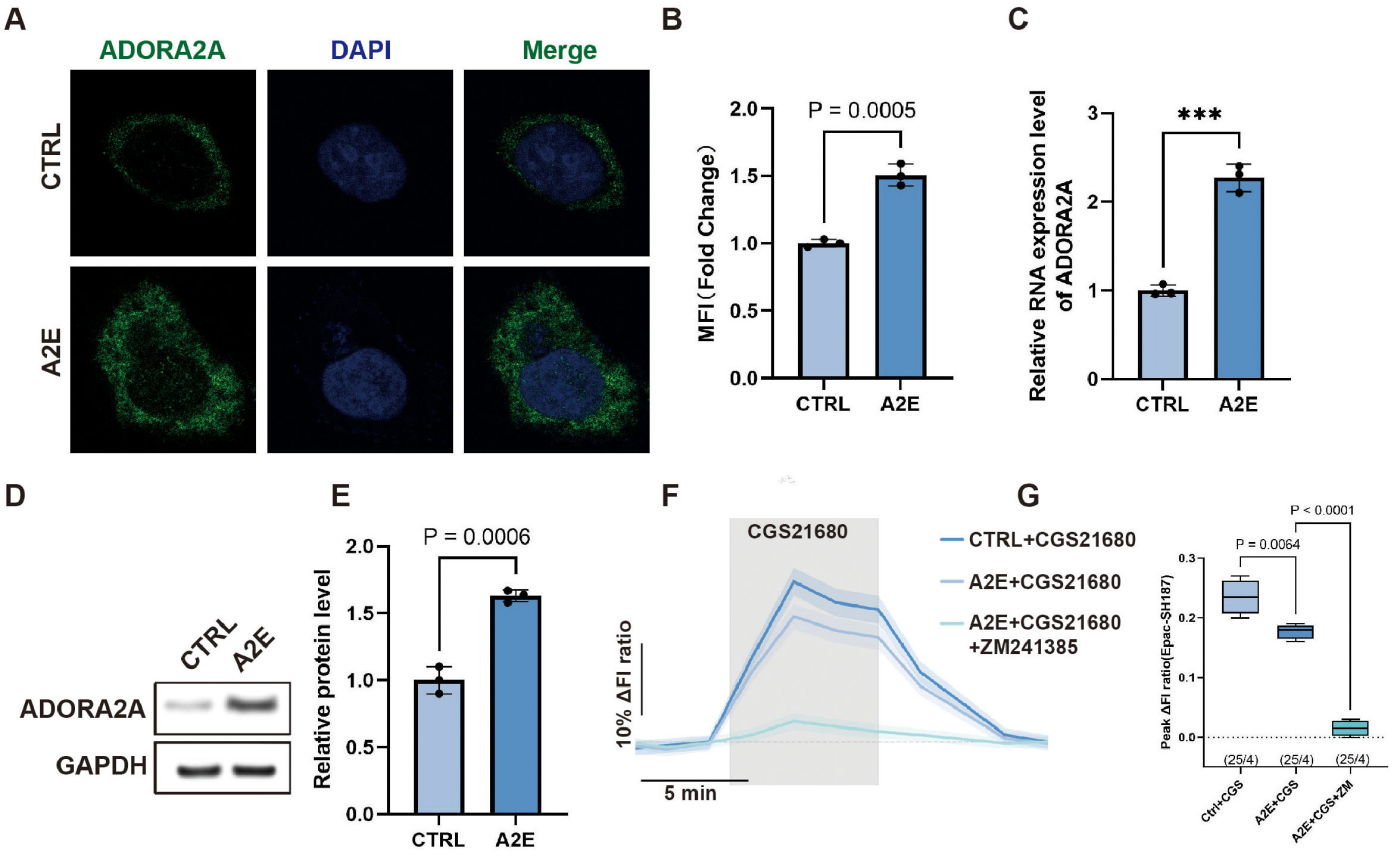
ADORA2A is upregulated and remains pharmacologically activatable in A2E-stressed RPE cells. **(A)** Representative immunofluorescence images of ADORA2A (green) and DAPI (blue) in control and A2E-treated ARPE-19 cells. Merge images are shown in the right column. **(B)** Quantification of ADORA2A mean fluorescence intensity (MFI) from images shown in **(A, C)** Relative mRNA expression of ADORA2A measured by quantitative PCR in control and A2E-treated ARPE-19 cells. **(D)** Representative immunoblot of ADORA2A protein expression in control and A2E-treated cells, with GAPDH as a loading control. **(E)** Densitometric quantification of ADORA2A protein levels shown in **(D, F)** Representative cAMP FRET traces in Epac-SH187-expressing ARPE-19 cells stimulated with CGS21680 under control and A2E-treated conditions, with or without ZM241385 pretreatment. The shaded region indicates CGS21680 application. **(G)** Quantification of peak cAMP responses from cells shown in **(F)** Box plots show the median, interquartile range, and whiskers. Numbers in parentheses indicate cells/independent experiments. Exact P values are shown in the figure. ***P<0.001.

To determine whether ADORA2A is altered under AMD-relevant stress conditions, ARPE-19 cells were exposed to A2E and analyzed at the protein and transcript levels. Immunofluorescence staining showed that ADORA2A immunoreactivity was increased in A2E-treated cells compared with control cells, with stronger and more widespread signal in the perinuclear and peripheral cytoplasmic regions (**Figure 1A**). Quantification of mean fluorescence intensity confirmed a significant increase in ADORA2A signal after A2E treatment (**Figure 1B**). Consistent with this finding, quantitative PCR showed that ADORA2A mRNA expression was significantly elevated in the A2E group (**Figure 1C**). Immunoblot analysis further demonstrated increased ADORA2A protein abundance in A2E-treated cells, and densitometric analysis confirmed this change (**Figures 1D, E**). Together, these results indicate that ADORA2A undergoes stress-responsive upregulation in RPE cells under A2E-induced lysosomal stress.

We next asked whether the receptor remained functionally activatable under these conditions. Real-time cAMP FRET imaging using the Epac-SH187 biosensor showed that CGS21680 induced a rapid increase in cAMP signal in control cells and also elicited a clear response in A2E-treated cells, although the peak response was modestly reduced relative to control cells (**Figure 1F**). Pretreatment with the A2A receptor antagonist ZM241385 markedly blunted this response, reducing the signal to near-baseline levels (**Figures 1F, G**). These findings indicate that although A2E stress remodels ADORA2A expression, ADORA2A-dependent cAMP signaling remains inducible and pharmacologically targetable in stressed RPE cells.

### 3.2 ADORA2A activation preferentially improves post-ingestive degradation of POS

**Figure 2:**
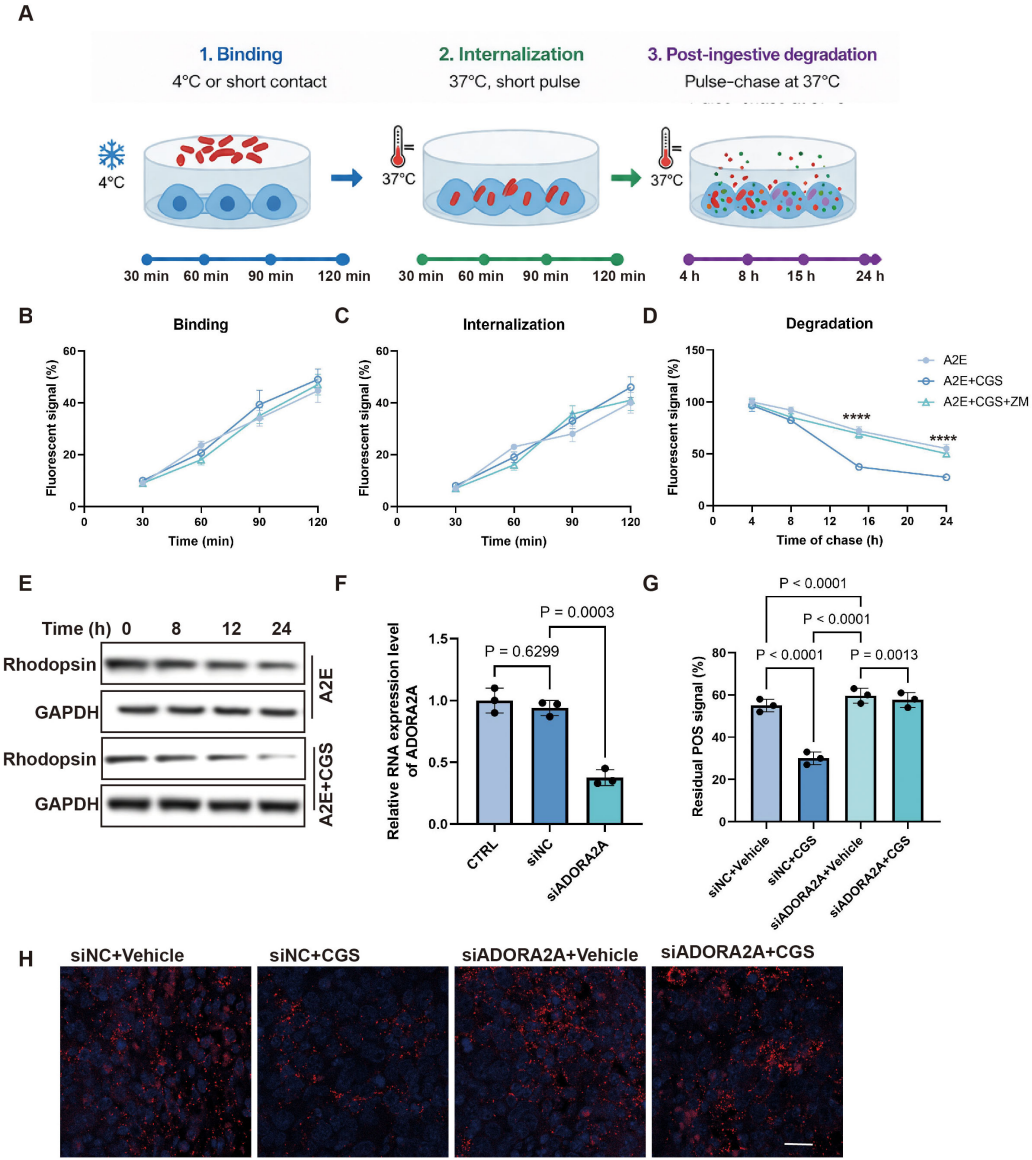
ADORA2A activation promotes post-ingestive POS processing and lysosomal function. **(A)** Experimental scheme illustrating the sequential assessment of POS handling in ARPE-19 cells, including binding, internalization, and post-ingestive degradation. Binding was assessed under 4 °C or short-contact conditions, internalization under a short pulse at 37 °C, and post-ingestive degradation by pulse-chase analysis at 37 °C. **(B)** Binding kinetics of TR-POS under A2E-treated conditions in the presence of vehicle, CGS21680, or CGS21680 plus ZM241385. Fluorescent signal is plotted over time. **(C)** Internalization kinetics of TR-POS under the indicated conditions. Internalized POS signal was quantified after exclusion of extracellular fluorescence. **(D)** Post-ingestive degradation kinetics assessed by pulse-chase analysis. Residual POS signal was measured at the indicated chase time points. **(E)** Representative immunoblots of rhodopsin and GAPDH during the POS pulse-chase degradation assay. ARPE-19 cells were chased for 0, 8, 12, and 24 h under A2E or A2E + CGS21680. **(F)** Knockdown efficiency of ADORA2A assessed by quantitative PCR in CTRL, siNC, and siADORA2A groups. Relative ADORA2A mRNA expression was normalized to the control condition. **(G)** Quantification of residual POS signal at 24 h chase in siNC + vehicle, siNC + CGS21680, siADORA2A + vehicle, and siADORA2A + CGS21680 groups. **(H)** Representative confocal images of residual intracellular POS at 24 h chase under the same conditions as in **(G)** Red, TR-POS; blue, DAPI. Scale bar, 50 µm. Data are presented as mean ± s.e.m. from independent experiments. Exact P values are shown in the figure where indicated. ****means p < 0.0001.

To determine which step of POS handling is primarily affected by ADORA2A signaling, we separated the process into three sequential phases: binding, internalization, and post-ingestive degradation (**Figure 2A**). In the early phase, TR-POS association under binding conditions increased over time in all groups. CGS21680 treatment produced only modest changes in the overall binding profile compared with the A2E+vehicle group, and this effect was not prominent across the time course (**Figure 2B**). A similar pattern was observed for internalization. Although CGS21680 showed a mild tendency to increase internalized POS signal at later early time points, the magnitude of this effect remained limited, and the CGS21680+ZM241385 group largely tracked close to the vehicle-treated condition (**Figure 2C**). These findings suggest that ADORA2A activation does not primarily act by markedly enhancing the initial association or uptake of POS. In contrast, a clearer divergence emerged during the pulse-chase phase. Residual POS signal declined progressively in all groups, but CGS21680 accelerated this decline, particularly at later chase time points, indicating more efficient post-ingestive degradation of internalized cargo (**Figure 2D**). This effect was attenuated by ZM241385, supporting the specificity of the response to ADORA2A signaling. To determine whether this reduction primarily reflects intracellular degradation rather than altered trafficking, we performed a time-course rhodopsin degradation assay by Western blotting. Rhodopsin, the most abundant POS protein, was tracked at 0, 8, 12, and 24 hours of chase. A2E-stressed cells exhibited delayed rhodopsin clearance, whereas CGS21680 treatment accelerated the degradation kinetics (**Figure 2E**). These results indicate that the principal effect of ADORA2A activation occurs after POS ingestion, promoting proteolysis of internalized cargo rather than simply increasing initial uptake.

We next asked whether this effect depended on ADORA2A expression. Quantitative PCR confirmed effective knockdown of ADORA2A by siRNA, whereas the siNC group showed no detectable change compared with the control condition (**Figure 2F**). Consistent with the pharmacologic data, CGS21680 significantly reduced residual POS signal at 24 h chase in siNC-transfected cells. By contrast, this reduction was largely lost after ADORA2A knockdown, and residual POS levels in the siADORA2A + CGS21680 group remained close to those in the siADORA2A+vehicle group (**Figure 2G**). Representative confocal images showed the same pattern, with visibly reduced intracellular POS retention in the siNC + CGS21680 group, whereas persistent POS accumulation remained evident after ADORA2A silencing despite CGS21680 treatment (**Figure 2H**). Together, these data indicate that ADORA2A activation preferentially enhances post-ingestive degradation of POS and that this effect is largely ADORA2A-dependent.

### 3.3 ADORA2A activation promotes post-ingestive POS processing and lysosomal function

**Figure 3:**
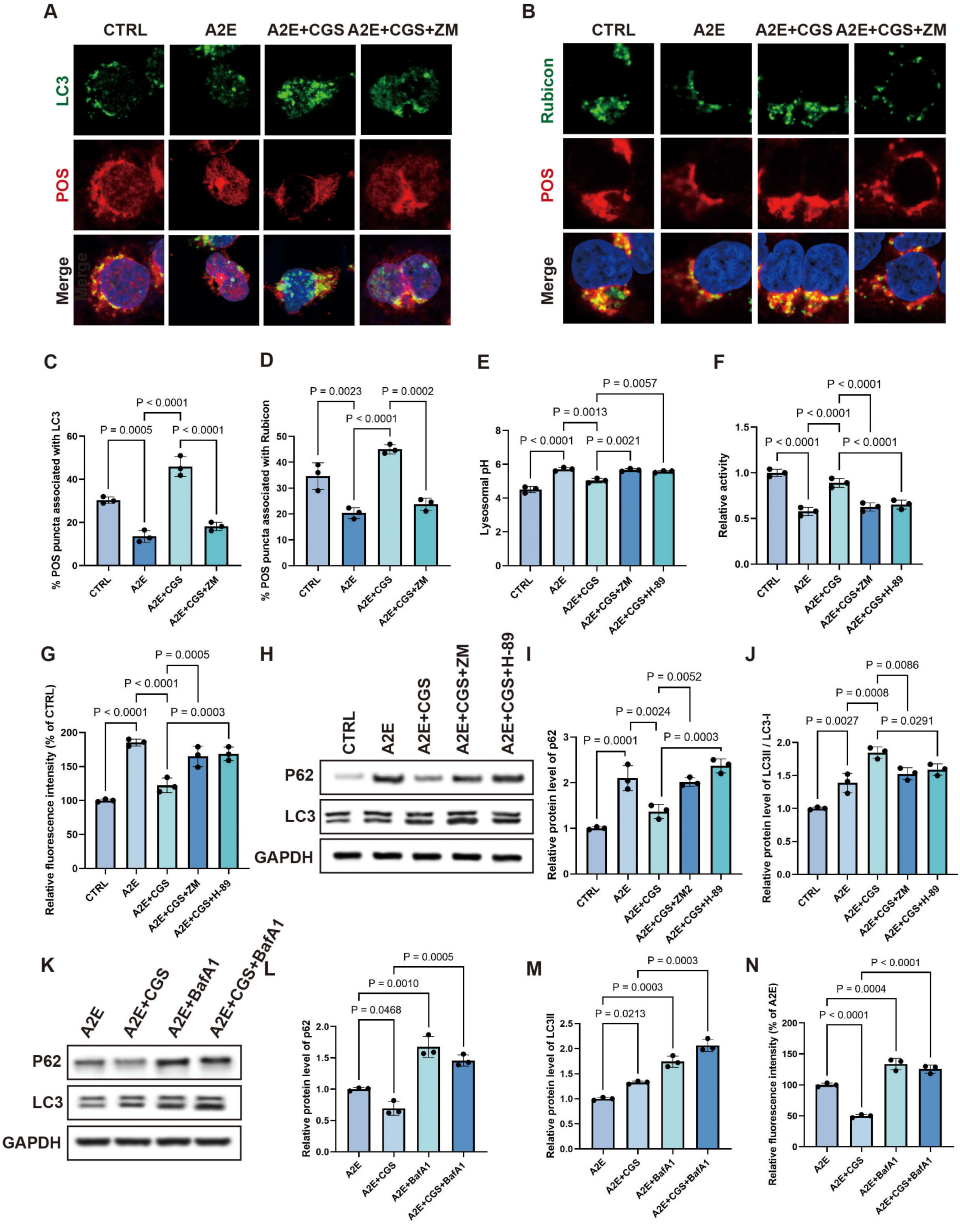
ADORA2A activation enhances LC3B- and Rubicon-associated POS processing and lysosome-dependent cargo turnover in A2E-stressed RPE cells. **(A)** Representative confocal images of LC3B (green), TR-POS (red), and DAPI (blue) in CTRL, A2E, A2E + CGS21680, and A2E + CGS21680 + ZM241385 groups. Merge images are shown in the right column, Scale bar, 50 µm. **(B)** Representative confocal images of Rubicon (green), TR-POS (red), and DAPI (blue) in CTRL, A2E, A2E + CGS21680, and A2E + CGS21680 + ZM241385 groups. Merge images are shown in the right column, Scale bar, 50 µm. **(C)** Quantification of the percentage of POS puncta associated with LC3 under the indicated conditions. **(D)** Quantification of the percentage of POS puncta associated with Rubicon under the indicated conditions. **(E)** Lysosomal pH measured by LysoSensor in the indicated groups. **(F)** Relative DQ Green BSA fluorescence intensity, shown as a readout of lysosomal proteolytic capacity. **(G)** Relative Cathepsin D activity in the indicated groups. **(H)** Representative immunoblots of p62, LC3, and GAPDH in CTRL, A2E, A2E + CGS21680, A2E + CGS21680 + ZM241385, and A2E + CGS21680 + H-89 groups. **(I)** Densitometric quantification of p62 protein levels normalized to GAPDH. **(J)** Quantification of the LC3II/LC3I ratio under the indicated conditions. **(K)** Representative immunoblots of p62, LC3B, and GAPDH in A2E-stressed RPE cells treated with CGS21680 and/or bafilomycin A1. **(L)** Quantification of p62 protein levels normalized to GAPDH in the bafilomycin A1 flux assay. **(M)** Quantification of LC3-II protein abundance normalized to GAPDH in the bafilomycin A1 flux assay. **(N)** Residual intracellular POS fluorescence intensity in A2E-stressed RPE cells treated with CGS21680 and/or bafilomycin A1, expressed relative to the A2E group. Data are presented as mean ± s.e.m. from at least three biologically independent experiments. Exact P values are shown in the figure. Statistical comparisons were performed using one-way ANOVA followed by Tukey’s multiple-comparisons test.

Having shown in **Figure 2** that ADORA2A activation primarily improves post-ingestive POS clearance rather than early POS uptake, we next examined whether this response was associated with altered recruitment of LC3B and Rubicon to POS-containing structures. Under basal conditions, a measurable proportion of internalized POS puncta was associated with LC3B and Rubicon. A2E exposure significantly reduced the association of POS puncta with both LC3B and Rubicon. In contrast, CGS21680 markedly increased LC3B and Rubicon association with POS-containing structures under A2E stress, whereas ZM241385 substantially attenuated these effects (**Figures 3A–D**). These findings indicate that ADORA2A activation promotes LC3- and Rubicon-associated processing of internalized POS. We next assessed whether these cargo-associated changes were accompanied by alterations in LC3B/p62 signaling and lysosomal degradative function. Immunoblot analysis showed that A2E exposure increased p62 abundance, whereas CGS21680 reduced p62 levels. Co-treatment with either ZM241385 or the PKA inhibitor H-89 attenuated the CGS21680-associated reduction in p62 (**Figures 3H, I**). In parallel, the LC3-II/LC3-I ratio was increased by CGS21680 relative to A2E treatment alone, and this increase was reduced by ZM241385 or H-89 (**Figures 3H, J**). Consistent with these molecular changes, A2E stress increased lysosomal pH and reduced Cathepsin D activity. CGS21680 lowered lysosomal pH toward the control range and restored Cathepsin D activity, whereas ZM241385 and H-89 attenuated these effects (**Figures 3E, F**). A2E also increased residual intracellular POS fluorescence following the chase period, indicating impaired post-ingestive POS clearance. CGS21680 significantly reduced residual POS fluorescence, while ZM241385 and H-89 diminished this improvement (**Figure 3G**).

To distinguish lysosome-dependent turnover from static changes in LC3B and p62 abundance, we next performed a bafilomycin A1 flux assay under A2E stress. Bafilomycin A1 increased p62 abundance and LC3-II accumulation in both A2E-treated and CGS21680-treated cells (**Figures 3K–M**). Importantly, bafilomycin A1 also significantly increased residual intracellular POS fluorescence in CGS21680-treated cells, indicating that inhibition of lysosomal degradation impaired the CGS21680-associated enhancement of POS clearance (**Figure 3N**). Collectively, these data indicate that ADORA2A activation enhances Rubicon- and LC3B-associated processing of internalized POS and improves lysosome-dependent cargo turnover in A2E-stressed RPE cells.

### 3.4 ADORA2A activation restores degradative machinery

**Figure 4:**
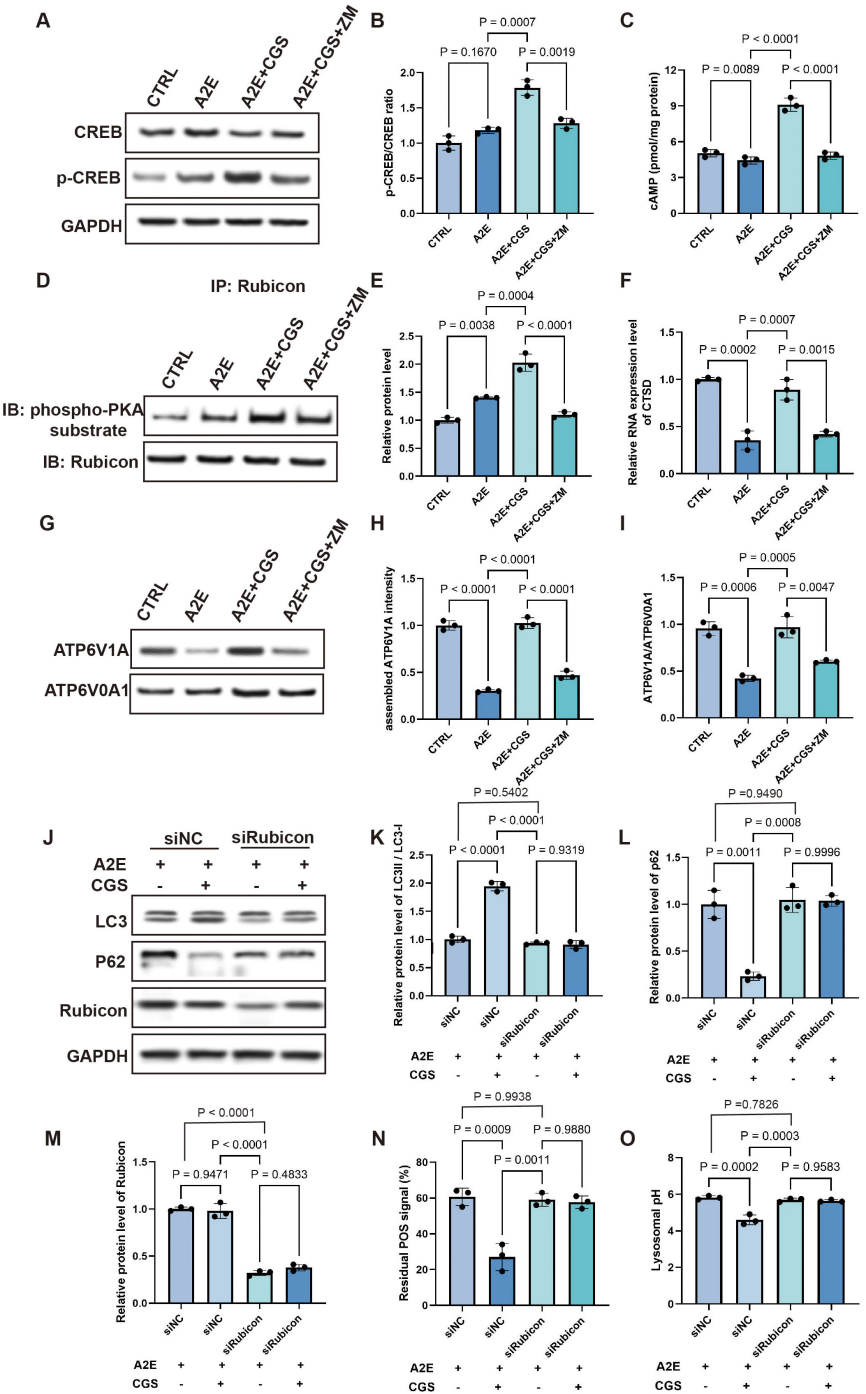
ADORA2A activation engages cAMP–CREB signaling and restores degradative machinery. **(A)** Representative immunoblots of CREB, phospho-CREB (p-CREB), and GAPDH in CTRL, A2E, A2E + CGS21680, and A2E + CGS21680 + ZM241385 groups. **(B)** Quantification of the p-CREB/CREB ratio under the indicated conditions. **(C)** Intracellular cAMP levels measured by ELISA in the indicated groups. **(D)** Rubicon immunoprecipitation followed by immunoblotting with phospho-PKA substrate antibody and Rubicon antibody. **(E)** Quantification of phospho-PKA substrate signal associated with immunoprecipitated Rubicon, normalized to immunoprecipitated Rubicon. **(F)** Relative mRNA expression of CTSD measured by quantitative PCR in the indicated groups. **(G)** Representative native PAGE/immunoblot showing assembly-associated ATP6V1A and ATP6V0A1 signals in lysosome-enriched fractions under the indicated conditions. **(H)** Quantification of assembled ATP6V1A intensity. **(I)** Quantification of the assembled ATP6V1A/ATP6V0A1 ratio. **(J)** Representative Western blots showing the protein expression of LC3, p62, and Rubicon. GAPDH was used as the loading control. **(K–M)** Densitometric quantification of the LC3-II/LC3-I ratio **(K)**, relative p62 protein level **(L)**, and relative Rubicon protein level indicating knockdown efficiency **(M)**. **(N)** Quantification of residual POS signal following the pulse-chase degradation assay. **(O)** Lysosomal pH measured using LysoSensor under the indicated siRNA and treatment conditions. Data are presented as mean ± s.e.m. from independent experiments. Exact P values are shown in the figure.

To further define how ADORA2A signaling connects to the restoration of post-ingestive degradative processing, we next examined upstream cAMP-responsive signaling and downstream degradative machinery. Under A2E-stressed conditions, the p-CREB/CREB ratio was increased by CGS21680 compared with A2E alone, whereas this increase was attenuated by ZM241385 (**Figures 4A, B**). Consistent with this, cAMP ELISA showed that intracellular cAMP levels were markedly elevated after CGS21680 treatment and returned toward baseline in the presence of ZM241385 (**Figure 4C**). These findings indicate that ADORA2A remains functionally coupled to cAMP–CREB signaling in A2E-stressed RPE cells. We then asked whether this signaling response is linked to Rubicon-associated remodeling. Immunoprecipitation of Rubicon followed by immunoblotting with a phospho-PKA substrate antibody showed that the phospho-PKA substrate signal associated with immunoprecipitated Rubicon was enhanced by CGS21680 and reduced by ZM241385 (**Figures 4D, E**). While these data highlight the presence of enhanced PKA-associated phosphorylation events within the Rubicon complex, they strongly suggest a functional linkage between upstream cAMP signaling and Rubicon remodeling. In parallel, A2E stress suppressed CTSD mRNA expression, whereas CGS21680 partially restored CTSD transcript abundance; this effect was again attenuated by ZM241385 (**Figure 4F**). These results suggest that ADORA2A activation is associated with PKA-sensitive Rubicon-related signaling and with transcriptional recovery of a key lysosomal protease gene. Because **Figure 3** showed that ADORA2A activation restores lysosomal acidification and degradative capacity, we finally tested whether this response is accompanied by recovery of V-ATPase assembly. Native PAGE analysis of lysosome-enriched fractions showed that A2E reduced assembled ATP6V1A signal, whereas CGS21680 restored this signal and ZM241385 attenuated the restoration (**Figures 4G, H**). Quantification of the assembled ATP6V1A/ATP6V0A1 ratio showed the same overall pattern, with a reduced ratio in A2E-treated cells, recovery after CGS21680 treatment, and partial loss of this recovery in the presence of ZM241385 (**Figure 4I**). Together, these data indicate that ADORA2A activation engages cAMP-responsive signaling, enhances Rubicon-associated PKA-substrate signaling, and restores V-ATPase-associated degradative machinery, thereby extending the mechanistic link between receptor activation and improved POS clearance.

To assess the functional contribution of Rubicon to ADORA2A-mediated recovery, Rubicon was depleted by siRNA in A2E-stressed ARPE-19 cells. Immunoblotting confirmed efficient reduction of Rubicon protein abundance (**Figures 4J, M**). In siNC-transfected cells, CGS21680 increased the LC3-II/LC3-I ratio and reduced p62 abundance. These CGS21680-associated changes were markedly attenuated following Rubicon depletion (**Figures 4J–L**). Consistent with these biochemical findings, CGS21680 reduced residual intracellular POS fluorescence in siNC cells, whereas this improvement was substantially impaired in siRubicon-treated cells (**Figure 4N**). Similarly, the CGS21680-associated reduction in lysosomal pH was attenuated after Rubicon depletion (**Figure 4O**). Together, these findings indicate that Rubicon is an important downstream contributor to the ADORA2A-associated restoration of LC3-linked POS processing and lysosomal function.

### 3.5 ADORA2A activation attenuates chronic stress–associated RPE dysfunction

**Figure 5:**
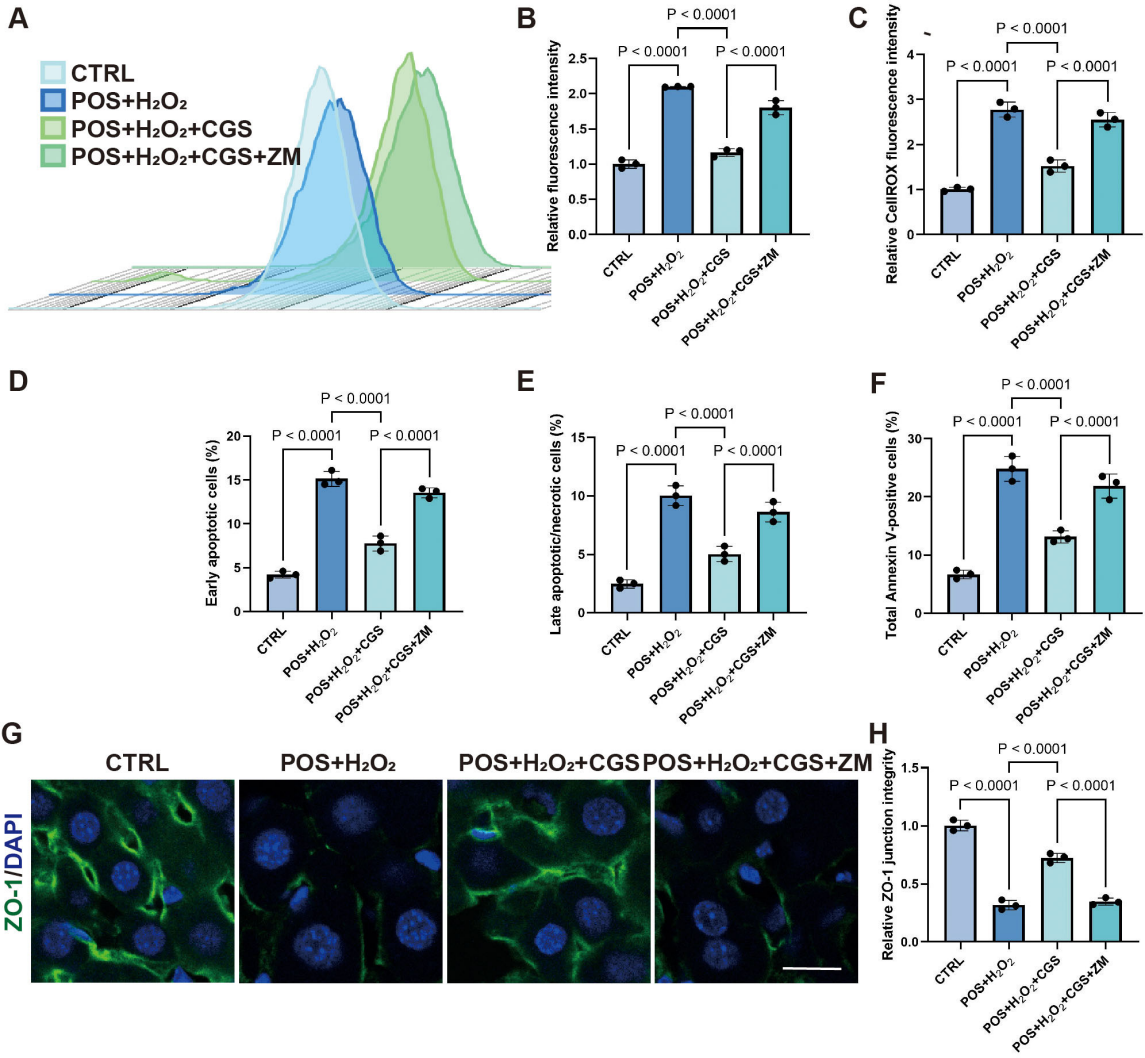
ADORA2A activation attenuates chronic stress–associated RPE dysfunction. Confluent ARPE-19 monolayers were challenged with POS once daily for 14 consecutive days and exposed during the same period to low-dose H_2_O_2_ to maintain chronic sublethal oxidative stress. CGS21680 and ZM241385 were added as indicated. **(A)** Representative flow-cytometric histograms of cellular autofluorescence in CTRL, A2E, A2E + CGS21680, and A2E + CGS21680 + ZM241385 groups. **(B)** Quantification of relative autofluorescence intensity. **(C)** Quantification of relative CellROX fluorescence intensity as a readout of intracellular ROS. **(D)** Quantification of early apoptotic cells (Annexin V-positive/PI-negative). **(E)** Quantification of total Annexin V-positive cells. **(F)** Quantification of late apoptotic/necrotic cells (Annexin V-positive/PI-positive). **(G)** Representative immunofluorescence images of ZO-1 (green) and DAPI (blue) under the indicated conditions. Scale bar, 10 µm. **(H)** Quantification of relative ZO-1 junction integrity. Data are presented as mean ± s.e.m. from independent experiments. Exact P values are shown in the figure.

In a chronic stress model based on daily POS challenge combined with low-dose H_2_O_2_ exposure for 14 days, we next asked whether restoration of post-ingestive degradative fitness by ADORA2A activation translates into protection against AMD-like cumulative injury. Flow-cytometric analysis showed that chronic stress markedly increased cellular autofluorescence, consistent with progressive accumulation of autofluorescent material accumulation, whereas CGS21680 reduced this increase. This protective effect was attenuated by ZM241385 (**Figures 5A, B**). A similar pattern was observed for intracellular oxidative stress. CellROX fluorescence was markedly elevated under chronic stress, decreased after CGS21680 treatment, and increased again in the presence of ZM241385 (**Figure 5C**). These findings indicate that ADORA2A activation reduces chronic stress–associated accumulation and oxidative burden. We then examined whether this response is accompanied by reduced cell injury. Chronic stress significantly increased the proportion of early apoptotic cells and the overall Annexin V-positive population, whereas CGS21680 attenuated both changes. ZM241385 again diminished this protective effect (**Figures 5D, E**). Late apoptotic/necrotic cells showed the same overall pattern, with increased cell death under chronic stress, partial rescue after CGS21680 treatment, and loss of this rescue in the presence of ZM241385 (**Figure 5F**). These data suggest that ADORA2A activation limits chronic stress–induced apoptotic injury in RPE cells. Finally, we asked whether these chronic stress conditions also affect epithelial integrity. Immunofluorescence staining showed that ZO-1 formed continuous junctional borders in control cells, whereas chronic stress disrupted junctional continuity and produced fragmented or weakened membrane-associated ZO-1 staining. CGS21680 partially restored ZO-1 continuity, and this improvement was attenuated by ZM241385 (**Figures 5G, H**). Together, these results indicate that ADORA2A activation not only improves degradative processing, but also attenuates chronic stress–associated autofluorescence, oxidative stress, cell injury, and junctional disruption in RPE cells.

### 3.6 ADORA2A activation preserves epithelial integrity and restores lysosomal proteolytic function in primary RPE cells under A2E stress

**Figure 6:**
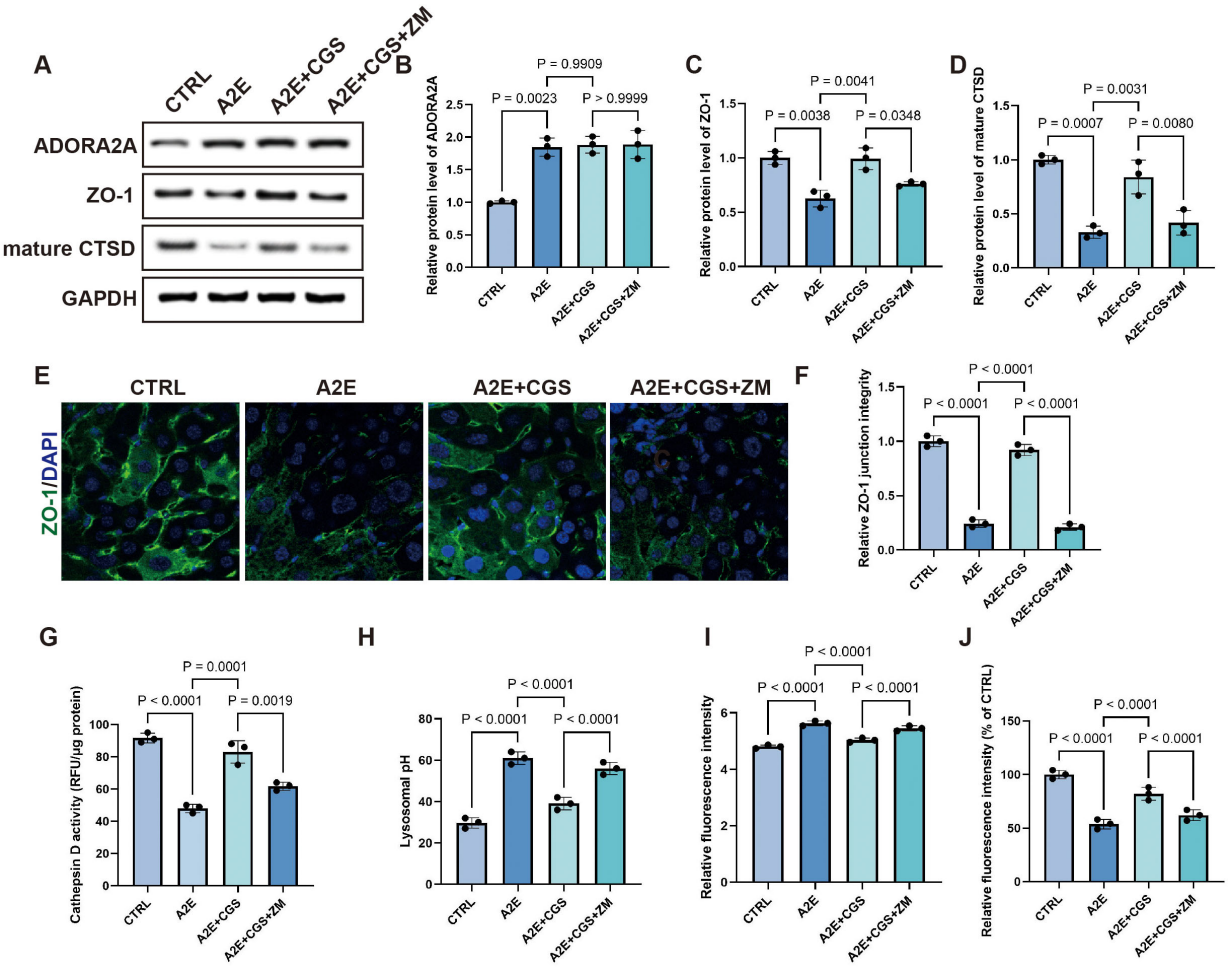
ADORA2A activation preserves epithelial integrity and lysosomal proteolytic competence in primary RPE cells under A2E stress. **(A)** Representative immunoblots of ADORA2A, ZO-1, mature Cathepsin D, and GAPDH in primary porcine RPE cells under CTRL, A2E, A2E + CGS21680, and A2E + CGS21680 + ZM241385 conditions. **(B)** Densitometric quantification of ADORA2A protein levels normalized to GAPDH. **(C)** Densitometric quantification of ZO-1 protein levels normalized to GAPDH. **(D)** Densitometric quantification of mature Cathepsin D protein levels normalized to GAPDH. **(E)** Representative immunofluorescence images of ZO-1 (green) and DAPI (blue) in the indicated groups. Scale bar, 20 μm. **(F)** Quantification of relative ZO-1 junction integrity. **(G)** Cathepsin D activity in primary porcine RPE cells under the indicated conditions, normalized to total protein content and expressed as RFU/μg protein. **(H)** Lysosomal pH measured by LysoSensor in the indicated groups. **(I)** Relative CellROX fluorescence intensity, shown as a readout of intracellular ROS. **(J)** Relative DQ Green BSA fluorescence intensity, shown as a readout of lysosomal proteolytic capacity (% of CTRL). Data are presented as mean ± s.e.m. from at least three independent experiments. Exact P values are shown in the figure. Statistical analysis was performed using one-way ANOVA followed by Tukey’s multiple-comparisons test. Exact P values are indicated in the figure.

To assess whether the protective effects of ADORA2A activation were reproducible in a more physiologically relevant system, we performed validation experiments in primary porcine RPE cells. Immunoblot analysis showed that ADORA2A protein abundance was significantly increased following A2E exposure compared with the CTRL condition. Neither CGS21680 treatment nor ZM241385 co-treatment materially altered this A2E-associated increase in total ADORA2A protein abundance (**Figures 6A, B**). In contrast, A2E exposure markedly reduced ZO-1 protein abundance, whereas CGS21680 significantly restored ZO-1 expression. This protective effect was attenuated by ZM241385 co-treatment (**Figures 6A, C**). Consistently, mature Cathepsin D (CTSD) abundance was substantially reduced under A2E stress, restored following CGS21680 treatment, and decreased again in the presence of ZM241385 (**Figures 6A, D**). These findings suggest that ADORA2A activation preserves both epithelial junction-associated protein expression and lysosome-associated protease maturation in primary RPE cells. Immunofluorescence staining further demonstrated that CTRL cells displayed relatively continuous ZO-1-positive intercellular junctions, whereas A2E disrupted junctional continuity and weakened membrane-associated ZO-1 staining. CGS21680 substantially restored ZO-1 junctional integrity, while ZM241385 abolished this improvement (**Figures 6E, F**). We next evaluated lysosomal proteolytic function and cellular stress phenotypes. Consistent with the reduction in mature CTSD abundance, Cathepsin D enzymatic activity was markedly decreased after A2E treatment. CGS21680 significantly restored CTSD activity, whereas ZM241385 attenuated this recovery (**Figure 6G**). A2E also increased lysosomal pH, indicating impaired lysosomal acidification; CGS21680 lowered lysosomal pH toward the CTRL range, and this effect was weakened by ZM241385 (**Figure 6H**). In parallel, intracellular ROS levels were elevated by A2E exposure, reduced by CGS21680 treatment, and increased again following ZM241385 co-treatment (**Figure 6I**). Finally, DQ Green BSA fluorescence, reflecting lysosomal proteolytic capacity, was reduced under A2E stress and partially restored by CGS21680; this improvement was attenuated by ZM241385 (**Figure 6J**). Collectively, these findings demonstrate that ADORA2A activation preserves epithelial junctional integrity, improves lysosomal acidification and proteolytic competence, and mitigates oxidative stress in primary RPE cells exposed to A2E.

### 3.7 ADORA2A activation preserves the outer blood-retinal barrier and visual function following acute RPE oxidative injury

**Figure 7:**
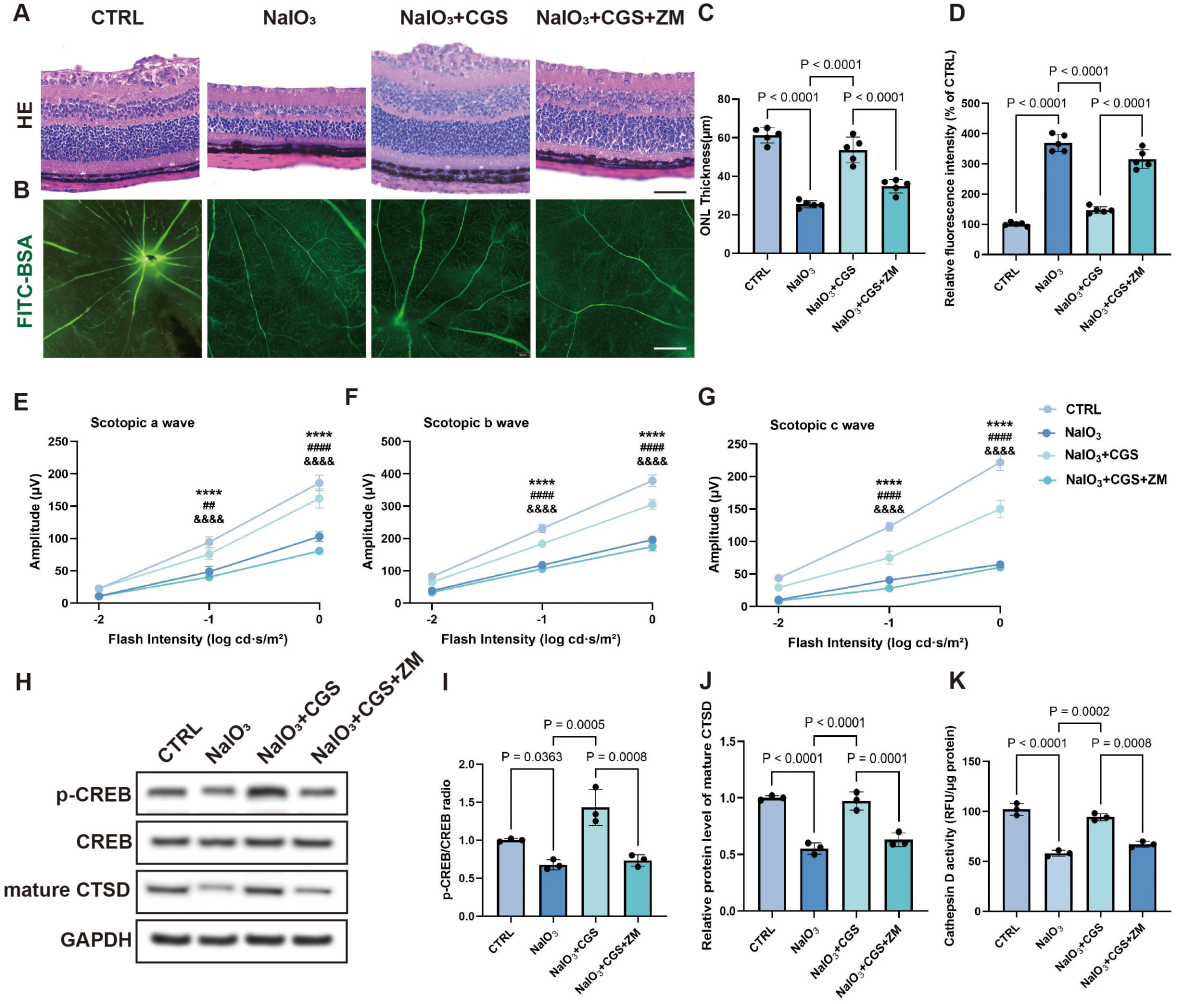
ADORA2A activation protects against acute retinal injury and restores lysosome-associated proteolytic competence *in vivo*. **(A)** Representative images of H&E-stained retinal sections Scale bars, 50μm. **(B)** FITC-BSA retinal flat mounts (bottom). Scale bars, 100 μm. **(C)** Quantification of outer nuclear layer (ONL) thickness (n= 5 eyes/group). **(D)** Quantification of relative FITC-BSA fluorescence intensity in retinal flat mounts (n = 5 eyes/group). **(E–G)** Quantitative analysis of scotopic a-wave **(E)**, b-wave **(F)**, and c-wave **(G)** amplitudes at varying flash intensities (-2, -1, and 0 log cd·s/m²) (n = 5 mice/group). **(H)** Representative immunoblots of p-CREB, total CREB, mature Cathepsin D (CTSD), and GAPDH in retinal lysates from the indicated groups. **(I)** Quantification of the p-CREB/CREB ratio. **(J)** Quantification of mature CTSD abundance normalized to GAPDH. **(K)** Cathepsin D enzymatic activity in retinal lysates, normalized to total protein content and expressed as RFU/μg protein. For panels I-K, n = 3 biologically independent retinal lysate samples per group. Data are presented as mean ± s.e.m. Exact P values are indicated in panels **(C, D, I–K)**. In **(E–G)**, significance at the corresponding flash intensity is indicated as follows: ****P < 0.0001, NaIO_3_ versus CTRL; ^####^P < 0.0001, NaIO_3_ + CGS21680 versus NaIO_3_; ^&&&&^P < 0.0001, NaIO_3_ + CGS21680 + ZM241385 versus NaIO_3_ + CGS21680.

To determine whether the protective effects of ADORA2A activation observed in stressed RPE cells translate to an *in vivo* setting, we employed a NaIO_3_-induced mouse model of acute retinal pigment epithelium injury. H&E staining showed that NaIO_3_ administration caused marked outer retinal degeneration, characterized by substantial thinning of the outer nuclear layer (ONL). Treatment with the ADORA2A agonist CGS21680 significantly preserved ONL thickness and attenuated photoreceptor loss, whereas co-treatment with the ADORA2A antagonist ZM241385 weakened this structural protection (**Figures 7A, C**). Because RPE integrity is essential for maintenance of the outer blood-retinal barrier, retinal leakage was further evaluated using FITC-BSA flat mounts. NaIO_3_ markedly increased retinal FITC-BSA fluorescence, indicating disruption of barrier integrity. CGS21680 significantly reduced FITC-BSA leakage, whereas ZM241385 attenuated the protective effect of CGS21680 (**Figures 7B, D**). We next examined retinal function using scotopic electroretinography. NaIO_3_ injury significantly reduced a-wave, b-wave, and c-wave amplitudes across flash intensities. CGS21680 treatment partially restored all three ERG parameters, whereas co-administration of ZM241385 markedly attenuated these functional improvements (**Figures 7E–G**). These findings indicate that ADORA2A activation protects retinal structure, outer blood-retinal barrier integrity, and visual function in NaIO_3_-injured mice. We then assessed downstream signaling and lysosome-associated proteolytic competence in retinal lysates from the indicated groups by western blotting and enzymatic assay. NaIO_3_ injury reduced the p-CREB/CREB ratio, whereas CGS21680 markedly increased CREB phosphorylation; this effect was attenuated by ZM241385 (**Figures 7H,I**). In parallel, mature Cathepsin D abundance was reduced following NaIO_3_ exposure, restored by CGS21680 treatment, and decreased again after ZM241385 co-treatment (**Figures 7H, J**). Consistent with these protein-level findings, Cathepsin D enzymatic activity was markedly suppressed by NaIO_3_, substantially recovered after CGS21680 treatment, and significantly attenuated by ZM241385 (**Figure 7K**). Collectively, these findings extend the protective effects of ADORA2A activation from stressed RPE cells to acute retinal injury *in vivo*, with retinal protection accompanied by CREB activation and recovery of lysosome-associated proteolytic competence.

## 4 Discussion

Our study identifies ADORA2A as a stress-responsive regulator of post-ingestive homeostasis in the RPE. Under A2E-induced lysosomal stress, ADORA2A expression increased at both the transcript and protein levels, while the receptor remained coupled to cAMP signaling and responsive to pharmacological activation. These findings indicate that stressed RPE cells retain an inducible adenosine-signaling axis despite stress-associated remodeling (Borea et al., 2018). Functionally, ADORA2A activation exerted only modest effects on early POS binding and internalization but produced a more pronounced and reproducible improvement in post-ingestive POS clearance (Ma et al., 2023). The attenuation of this effect by receptor antagonism and ADORA2A depletion supports its receptor dependence. ADORA2A therefore appears to regulate degradative fitness after cargo entry rather than broadly enhancing initial phagocytic uptake (Zihni, 2025).

A central implication of these findings is that ADORA2A signaling functionally couples post-ingestive cargo processing to lysosomal recovery (Miller et al., 2025). A2E exposure reduced the association of LC3B and Rubicon with POS-containing structures, whereas CGS21680 enhanced these associations and ZM241385 attenuated the response. These changes were accompanied by restoration of lysosomal acidification, DQ Green BSA proteolysis, and Cathepsin D activity. Rather than correcting a single isolated defect, ADORA2A activation therefore appears to improve several coordinated components of the post-ingestive degradative pathway (He et al., 2025).

The bafilomycin A1 experiments further support the involvement of lysosome-dependent turnover. Bafilomycin A1 increased LC3-II and p62 accumulation in both vehicle- and CGS21680-treated cells under A2E stress, consistent with ongoing lysosomal turnover of these proteins. Bafilomycin A1 also increased residual intracellular POS fluorescence and markedly attenuated the CGS21680-associated improvement in POS clearance. These findings indicate that the effect of ADORA2A activation on post-ingestive cargo handling depends, at least in part, on functional lysosomal degradation. This organization is particularly relevant to AMD, in which defective cargo handling may reflect disruption across several interconnected post-ingestive steps rather than a single molecular lesion (Boya et al., 2023).

Our upstream findings connect this phenotype to cAMP-responsive signaling and recovery of lysosomal degradative machinery. CGS21680 increased intracellular cAMP levels and CREB phosphorylation under A2E stress, whereas ZM241385 attenuated both responses. CGS21680 also enhanced phospho-PKA-substrate reactivity within Rubicon immunoprecipitates and restored V-ATPase assembly-associated signals in lysosome-enriched fractions. Together, these observations support a model in which ADORA2A activation engages cAMP-responsive signaling and promotes recovery of lysosomal degradative machinery. However, the direct molecular events connecting these signaling and degradative components remain unresolved.

Rubicon is an important functional component of this response. Rubicon depletion markedly attenuated the CGS21680-associated increase in the LC3-II/LC3-I ratio, the reduction in p62 abundance, and the improvements in POS clearance and lysosomal acidification. These findings indicate that Rubicon contributes substantially to the tested effects of CGS21680 on LC3-associated post-ingestive POS processing and lysosomal recovery. This interpretation is consistent with the established role of Rubicon in LC3-associated phagocytic and lysosome-related pathways (Wong et al., 2018). Nevertheless, Rubicon should not be regarded as the sole downstream mediator of ADORA2A signaling, and additional pathways may contribute to the overall protective phenotype.

The broader protective effects observed under chronic stress conditions are consistent with this mechanistic framework. ADORA2A activation reduced autofluorescence, intracellular ROS, and apoptotic injury while partially preserving ZO-1-defined junctional integrity. These data suggest that improved post-ingestive degradative handling is associated with more general stabilization of RPE homeostasis (Sharma et al., 2021). The parallel improvement in POS clearance and epithelial junctional integrity is of particular interest, because junctional disorganization may represent a downstream consequence of persistent intracellular burden, oxidative stress amplification, and impaired lysosomal processing (Markitantova and Simirskii, 2025). Thus, restoration of post-ingestive degradative competence may help limit progression from intracellular stress to epithelial instability.

The validation experiments in primary porcine RPE cells further strengthen this interpretation by demonstrating that the protective pattern is not restricted to ARPE-19 cells. In primary RPE cells, CGS21680 restored ZO-1 abundance and junctional organization, improved lysosomal acidification, reduced ROS, and enhanced DQ Green BSA processing. Importantly, CGS21680 also restored mature Cathepsin D abundance and Cathepsin D enzymatic activity, providing additional evidence that ADORA2A activation improves lysosomal proteolytic competence in a more physiologically relevant RPE system (Markert et al., 2022). The *in vivo* findings extend these observations to acute retinal injury. In the NaIO_3_ model, CGS21680 preserved ONL thickness, reduced FITC-BSA leakage, and partially restored scotopic a-wave, b-wave, and c-wave responses. These structural, barrier, and functional improvements were accompanied by increased CREB phosphorylation and recovery of mature Cathepsin D abundance and enzymatic activity in retinal lysates. Collectively, these data indicate that ADORA2A-associated retinal protection *in vivo* is accompanied by recovery of lysosome-associated proteolytic competence. Although these biochemical measurements were obtained from retinal tissue rather than purified RPE cells, they support the relevance of a CREB-associated lysosomal response during acute retinal injury.

From a translational perspective, these findings suggest that selectively restoring post-ingestive processing may represent a rational therapeutic strategy. The major benefit of ADORA2A activation emerged after cargo entry, at the levels of LC3-associated processing, lysosomal acidification, and proteolysis. This distinction may be particularly relevant to early AMD, in which RPE cells are chronically stressed but are not yet irreversibly lost. The observation that ADORA2A is upregulated under stress while remaining pharmacologically responsive further suggests that the diseased state may retain a therapeutic window for receptor-targeted intervention.

Several limitations should be considered when interpreting these findings. Although the bafilomycin A1 experiments demonstrate that the CGS21680-associated changes in LC3-II, p62, and POS clearance involve lysosome-dependent turnover, bafilomycin A1 alone cannot distinguish LC3 lipidation on POS-containing phagosomes from canonical autophagy. The recruitment of LC3B and Rubicon to POS-containing structures, together with the loss of the CGS21680 response following Rubicon depletion, supports the involvement of Rubicon-dependent LC3-associated post-phagocytic processing. Nevertheless, canonical autophagy initiation was not independently examined. Perturbation of FIP200, ULK1, ATG13, or ATG14, together with ultrastructural analysis of phagosomal membrane topology, will be required to determine whether bona fide LC3-associated phagocytosis contributes to this phenotype. We therefore describe the observed process as Rubicon-dependent, lysosome-associated processing of internalized POS rather than definitive LAP.

The present data also support functional coupling between ADORA2A, cAMP-responsive signaling, and recovery of lysosomal degradative machinery, but do not define the direct molecular events connecting these components. Enhanced phospho-PKA-substrate reactivity in Rubicon immunoprecipitates does not establish Rubicon itself as a direct PKA substrate; instead, it indicates increased PKA-associated phosphorylation within the Rubicon-containing complex. Identifying the relevant phosphorylated protein(s) and modification sites will require targeted biochemical or phosphoproteomic analyses, particularly because post-translational modification of Rubicon has been reported to regulate Rubicon-associated pathways (Zambrano et al., 2019).

The translational and cellular specificity of these findings also requires further investigation. Primary-cell validation was performed in porcine RPE cells, whereas the *in vivo* biochemical analyses used whole-retinal lysates and therefore cannot attribute the observed changes specifically to the RPE. Confirmation in human donor-derived RPE cells and through RPE-specific *in vivo* approaches will be important. Moreover, ADORA2A signaling is highly context-dependent. The present study examines short-term pharmacological activation, whereas chronic or sustained ADORA2A signaling has been associated with divergent, including pro-inflammatory or pro-fibrotic, effects in other tissues. The protective effects observed here should therefore not be extrapolated to prolonged stimulation or other physiological contexts without additional investigation.

Despite these limitations, the present findings support a model in which ADORA2A links stress-responsive signaling to improved post-ingestive degradative capacity. By promoting Rubicon-dependent LC3-associated processing of internalized POS and improving lysosomal acidification, proteolytic competence, and epithelial integrity, ADORA2A activation may help preserve multiple aspects of RPE homeostasis relevant to early AMD pathogenesis.

## Data Availability

The original contributions presented in the study are included in the article/**Supplementary Material**, further inquiries can be directed to the corresponding author/s.
