# Supplementary material for: ADORA2A activation restores lysosomal function and photoreceptor outer segment degradation in stressed retinal pigment epithelium
Source: Front Physiol. 2026 Aug 5;17:1866784. doi: 10.3389/fphys.2026.1866784 (PMC13485735; doi:10.3389/fphys.2026.1866784)

Fig 1D

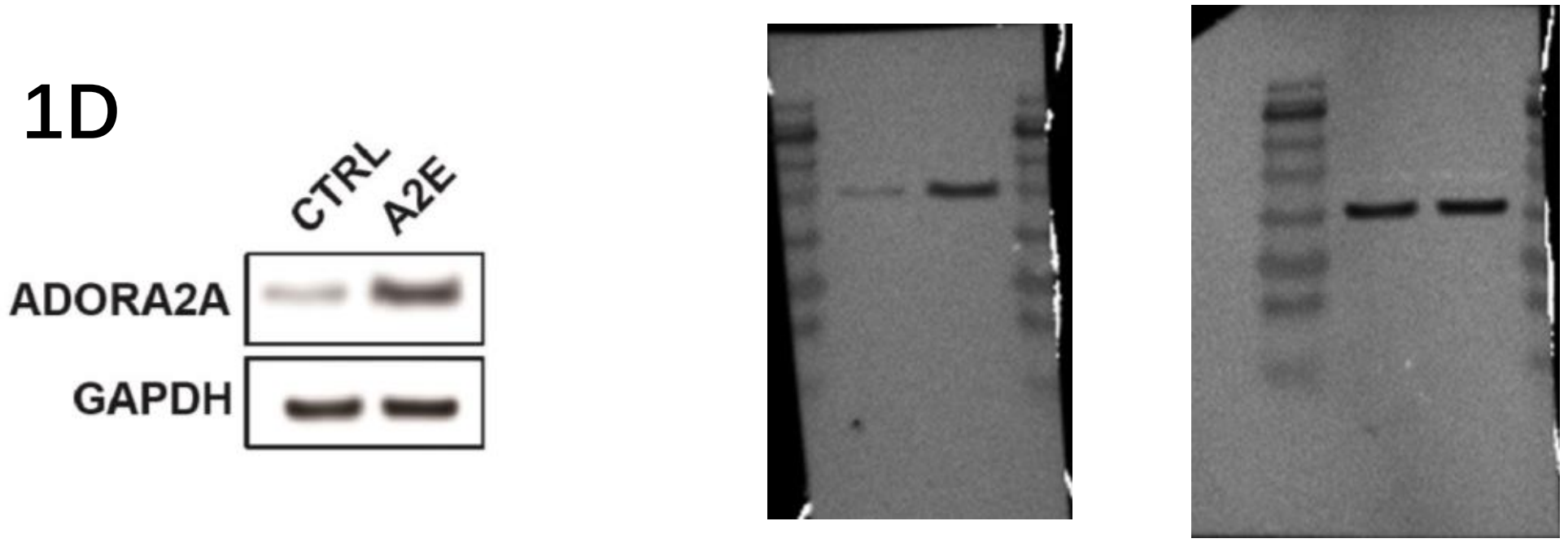

Fig 3E

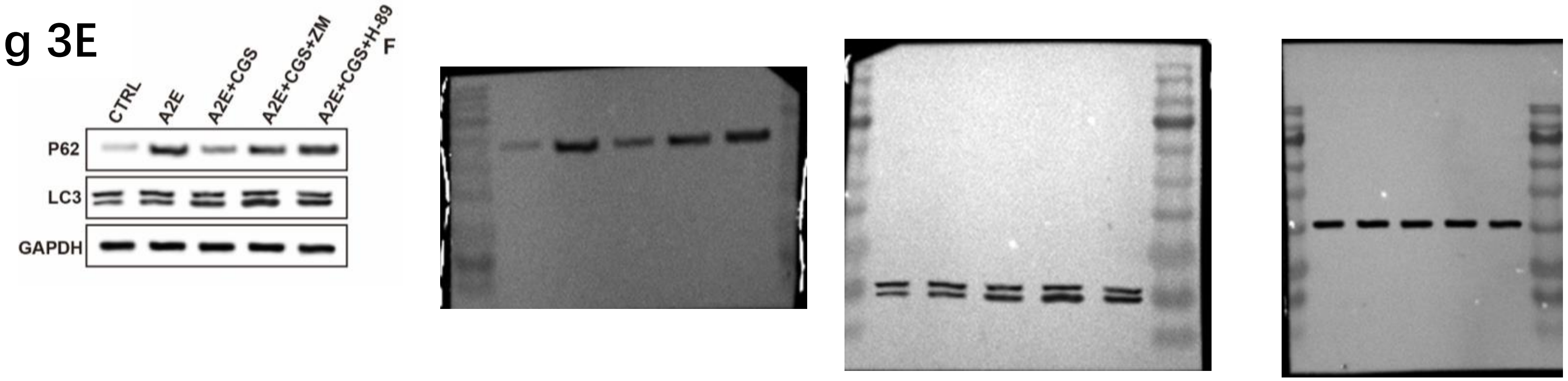

Fig 4A

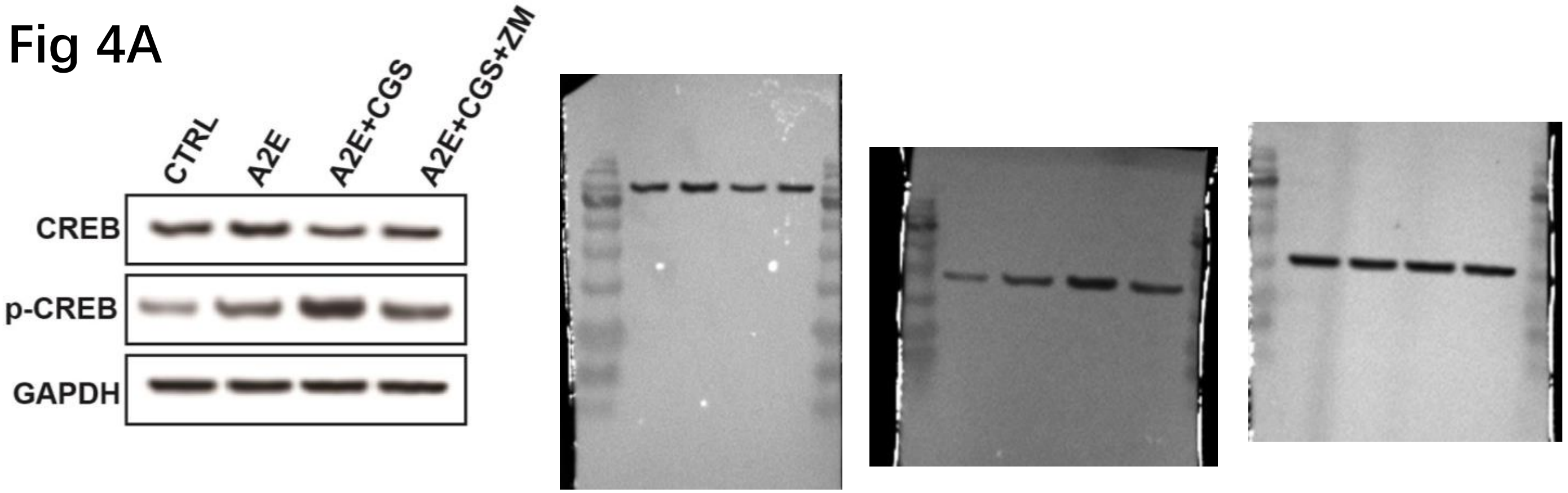

Fig 4D

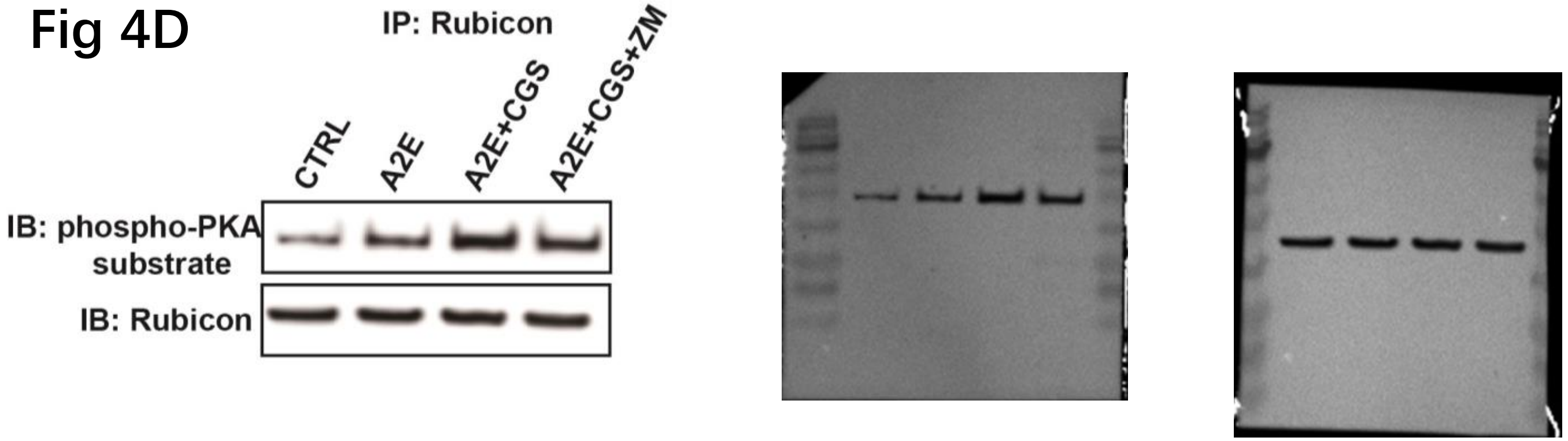

Fig 4G

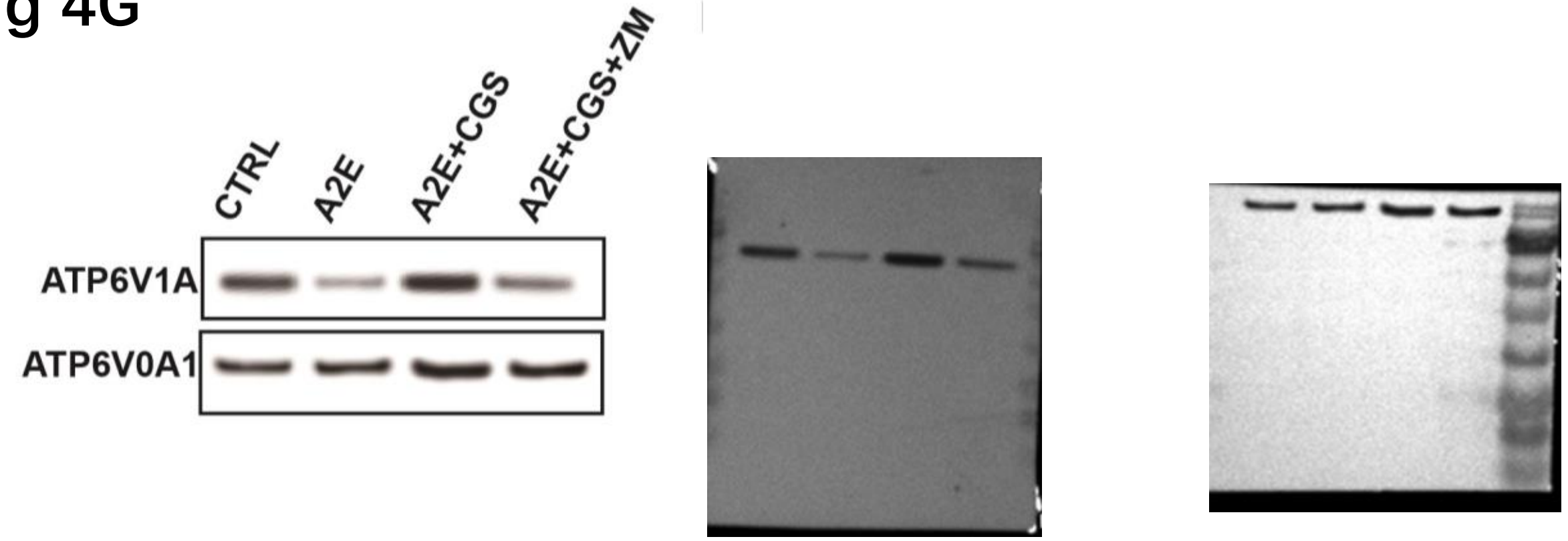

Fig 6A

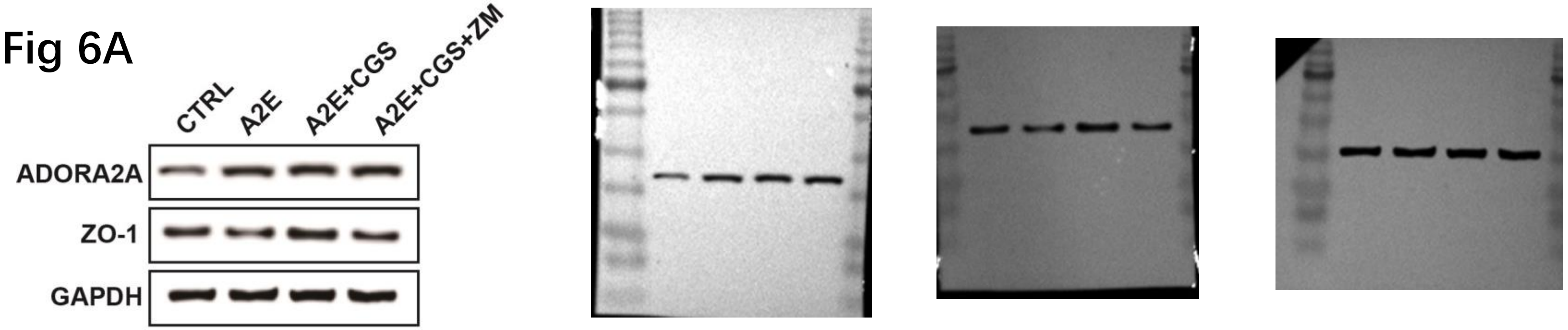

Supplement: Supplementary file 1 [file DataSheet1.pdf]
